# 超高效液相色谱-高分辨质谱法检验4种酰胺类合成大麻素同分异构体

**DOI:** 10.3724/SP.J.1123.2023.07007

**Published:** 2024-01-08

**Authors:** Ruiyang TANG, Jifen WANG, Tingyue XIAO, Shiyang QIN, Zhaowei JIE, Aolin ZHANG

**Affiliations:** 1.中国人民公安大学侦查学院, 北京 100038; 1. School of Investigation, People’s Public Security University of China, Beijing 100038, China; 2.北京市公安司法鉴定中心, 法庭毒物分析公安部重点实验室, 北京 100192; 2. Key Laboratory of Forensic Toxicology, Ministry of Public Security, Forensic Science Service of Beijing Public Security Bureau, Beijing 100192, China

**Keywords:** 超高效液相色谱-高分辨质谱, 合成大麻素, 同分异构体, ultra-high performance liquid chromatography-high resolution mass spectroscopy (UHPLC-HRMS), synthetic cannabinoids (SCs), isomers

## Abstract

同分异构现象在合成大麻素中普遍存在,因其结构与性质上的差异并不显著,当同时存在时分离鉴定较为困难,为公安实践中合成大麻素的检验鉴定带来了一定的挑战。本研究利用超高效液相色谱-高分辨质谱技术(UHPLC-HRMS)建立了5F-EMB-PICA与5F-MDMB-PICA、ADB-BINACA与AB-PINACA这2对酰胺类合成大麻素同分异构体的检验方法。选用Hypersil GOLD C_18_色谱柱(100 mm×2.1 mm, 1.9 μm)进行UHPLC分离,以含0.1%甲酸的甲醇溶液和含10 mmol/L甲酸铵的0.1%甲酸水溶液为流动相进行梯度洗脱。高分辨质谱采用一级质谱全扫描/数据依赖二级质谱扫描(Full MS/dd-MS^2^)进行检验。结果表明,采用上述仪器条件,能够实现4种合成大麻素同分异构体的分离分析,5F-EMB-PICA和5F-MDMB-PICA的分离度为2.06, ADB-BINACA和AB-PINACA的分离度为1.22,均达到了有效分离的目的。研究进一步开展了方法学指标考察,5F-EMB-PICA、5F-MDMB-PICA、ADB-BINACA和AB-PINACA这4种酰胺类合成大麻素同分异构体的线性关系均较好,相关系数(*R*^2^)均大于0.99。毛发中4种合成大麻素的基质效应范围为88.67%~111.76%,回收率为96.23%~105.11%,日内、日间精密度(RSD)均小于10%。使用本研究建立的检验方法鉴别案件检材,在毛发检材中检出了AB-PINACA,含量为0.73 μg/g;在烟丝检材中检出了5F-MDMB-PICA,含量为11.3 mg/g。研究结果表明所建方法可应用于公安机关对实际案件检材的检验,可为合成大麻素同分异构体的检验鉴定提供方法参考与思路借鉴。

## Abstract (en)

Isomerization commonly occurs in synthetic cannabinoids (SCs). Owing to the few differences in their structure and properties, it is difficult to simultaneously separate and identify them. Thus, the identification of synthetic cannabinoids is challenging, posing a threat to public security. This study aims to separate and identify four SCs, which are 2-[1-(5-fluoropentyl)-1*H*-indole-3-formylamino]-3,3-dimethylbutyrate methyl ester (5F-MDMB-PICA), 2-[1-(5-fluoropentyl)-1*H*-indole-3-formylamino]-3-methylbutyrate ethyl ester (5F-EMB-PICA), *N*-(1-amino-2,2-dimethyl-1-oxobutyl-2-yl)-1-butyl-1*H*-indazole-3-formamide (ADB-BINACA), *N*-(1-carbamoyl-2-methylpropyl)-1-pentyl indazole-3-formamide (AB-PINACA).Supercritical fluid chromatography-mass spectroscopy (SFC-MS) can realize the effective separation of some cannabinoid isomers. However, most laboratories are not equipped with SFC-MS systems. Ultra-high performance liquid chromatography-high resolution mass spectroscopy (UHPLC-HRMS) effectively combines the excellent efficient separation characteristics of liquid chromatography and the powerful qualitative ability of mass spectrometry. It is a commonly used technical method for the detection of amide synthetic cannabinoids and their metabolites in vivo and in vitro because of its advantages of high accuracy and efficiency. Liquid chromatography allows the separation of tested components by exploiting the difference in the partition coefficients between the mobile and stationary phases. When the two phases are in relative motion, the tested components are divided between the two phases, facilitating the separation and analysis of each component. Although the difference in the polarities of the tested amide synthetic cannabinoid isomeric substances is extremely small, liquid chromatography can induce a strong separation effect. The advantages of UHPLC-HRMS include high resolution imparted by mass spectrometry and high sensitivity, allowing its application in the qualitative analysis of various substances. Through UHPLC-HRMS, trace analytes at the nanogram scale as well as pure drugs and their metabolites in biosamples can be detected.

This study proposed a method for the determination of two pairs of amide synthetic cannabinoid isomers-5F-EMB-PICA and 5F-MDMB-PICA, ADB-BINACA and AB-PINACA-through UHPLC-HRMS. A Hypersil GOLD C_18_ column (100 mm×2.1 mm, 1.9 μm) was selected for separation via liquid chromatography, and gradient elution was performed with methanol containing 0.1% formic acid and a 0.1% formic acid aqueous solution containing 10 mmol/L ammonium formate. Full scan/data-dependent secondary mass spectrometry (Full MS/dd-MS^2^) was conducted in the positive ion mode for detection. The results indicated that the four synthetic cannabinoid isomers could be accurately detected under the abovementioned conditions. The resolution between 5F-EMB-PICA and 5F-MDMB-PICA was 2.06, while that between ADB-BINACA and AB-PINACA was 1.22, indicating the effective separation and detection of both pairs. Furthermore, method validation was conducted to ensure the accuracy of the proposed method. The relationship of the four amide synthetic cannabinoid isomers exhibited excellent linearity. The correlation coefficients (*R*^2^) were >0.99. Moreover, the matrix effects of the four SCs in hair samples were between 88.67% and 111.76% and the recoveries were 96.23%-105.11%. The intra-day and inter-day precisions (RSDs) were <10%. The proposed method was used to identify the case materials. AB-PINACA was detected in a hair sample at a content of 0.73 μg/g. 5F-MDMB-PICA was detected in a tobacco sample at a content of 11.3 mg/g. The results indicate that the proposed method can be used for the examination of practical samples conducted by public security organizations. This study provides a reference method for the identification of synthetic cannabinoid isomers.

合成大麻素类(synthetic cannabinoids, SCs)新精神活性物质是通过模拟大麻的主要精神活性成分四氢大麻酚(Δ^9^-THC)与内源性大麻素受体CB_1_结合而起兴奋作用^[1⇓-3]^,吸食后能产生甚于天然大麻的强烈快感,产生严重依赖性,继而出现滥用情况,是当今世界范围内种类最多、滥用最为严重的新精神活性物质之一。因其广泛滥用和娱乐致瘾的特性,目前SCs已成为全球共同关注的热点^[4⇓⇓-7]^。其中,*N*-(1-氨基-2,2-二甲基-1-氧代丁基-2-基)-1-丁基-1*H*-吲唑-3-甲酰胺(ADB-BINACA)、*N*-(1-氨甲酰基-2-甲基丙基)-1-戊基吲唑-3-甲酰胺(AB-PINACA)、2-[1-(5-氟戊基)-1*H*-吲哚-3-甲酰氨基]-3,3-二甲基丁酸甲酯(5F-MDMB-PICA)和2-[1-(5-氟戊基)-1*H*-吲哚-3-甲酰氨基]-3-甲基丁酸乙酯(5F-EMB-PICA)是近几年出现频率较高的酰胺类SCs。这4种酰胺类合成大麻素分子的结构信息见图1。

**图1:**
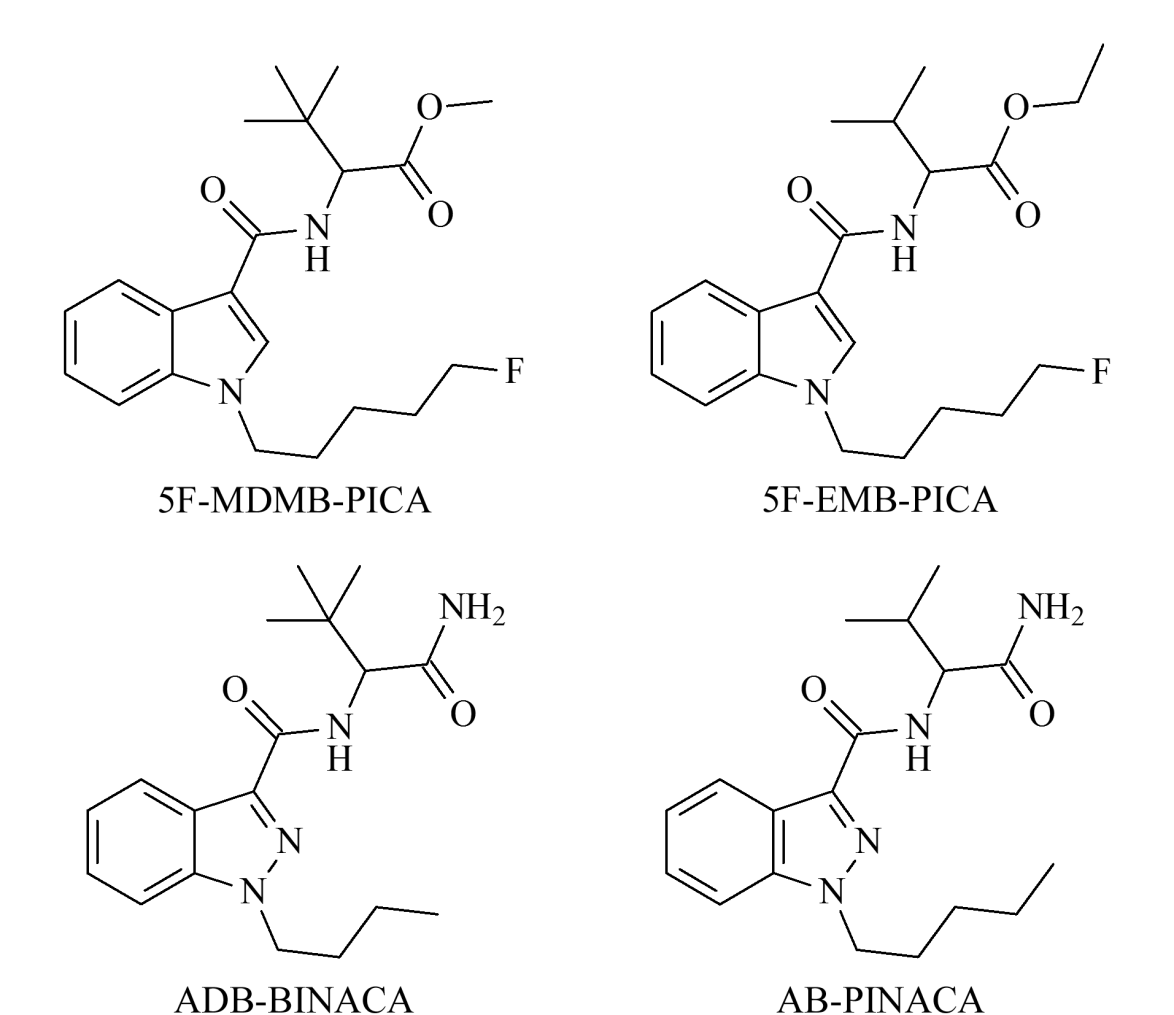
4种合成大麻素的结构信息

同分异构现象在合成大麻素类、合成卡西酮类、芬太尼类等新精神活性物质中普遍存在^[8,9]^。5F-MDMB-PICA与5F-EMB-PICA、ADB-BINACA与AB-PINACA这两对同分异构体均是由于一个甲基位置的不同而产生异构^[10⇓-12]^,因而在结构与性质上的差异较小,当同时存在时分离鉴定较为困难,给公安实践中该类SCs的检验鉴定带来了一定的困难与挑战。现有文献显示,检验酰胺类SCs的方法主要有气相色谱-质谱法(GC-MS)^[10]^、高效液相色谱-串联质谱法(HPLC-MS/MS)^[11]^、超高效液相色谱-高分辨质谱法(ultra-high performance liquid chromatography-high resolution mass spectroscopy, UHPLC-HRMS)^[12]^以及超临界流体色谱-质谱法(supercritical fluid chromatography-mass spectroscopy, SFC-MS)^[13]^等,而对于同分异构体的区分研究目前尚显缺乏。Toyo’oka等^[14]^利用SFC-MS方法成功鉴别出8种合成大麻素,采用CO_2_作为超临界流体色谱流动相,结合少量极性溶剂,实现了部分合成大麻素同分异构体的有效分离,效果理想。然而目前绝大部分法庭科学实验室都没有配备超临界流体色谱-质谱仪器,因而该技术方法并不具备公安实战推广价值。目前有关5F-MDMB-PICA与5F-EMB-PICA、ADB-BINACA与AB-PINACA同分异构体之间检验鉴定和有效区分的方法尚鲜有报道。

UHPLC-HRMS联用技术近年来发展迅速,是法医毒理学领域中检验SCs的热点应用技术。目前国内外将该技术广泛应用于多种SCs代谢机理以及方法研究^[15⇓-17]^。Tynon等^[18]^建立了血液中酰胺类SCs的LC-MS/MS快速检测方法,成功检出了AB-PINACA等8种合成大麻素;李佳瑞等^[4]^利用UHPLC-HRMS技术,借助斑马鱼实验模型,筛选出了5F-EMB-PICA的潜在代谢标志物,效果较为理想;古锟山等^[19]^采用LC-HRMS分析比较了斑马鱼体内代谢模型、人体尿液样本以及肝微粒体体外代谢模型以及人体真实尿液样本中ADB-BINACA的代谢情况。

本研究建立了有效区分和同时检测5F-MDMB-PICA与5F-EMB-PICA、ADB-BINACA与AB-PINACA两对SCs同分异构体的UHPLC-HRMS方法,并将所建立方法应用于实战案例的检材检验,旨在为实践中相关SCs同分异构体的检验鉴定提供思路参考和方法支撑。

## 1 实验部分

### 1.1 仪器、药品与试剂

Vanquish超高效液相色谱仪-Q Exactive^TM^ HF组合型四极杆Orbitrap质谱仪(美国赛默飞公司),配有可加热电喷雾离子源H-ESI。VORTEX2涡旋定速混匀器(德国IKA公司)。

### 1.2 药品与试剂

甲酸铵、甲酸和甲醇(色谱纯,纯度99.99%,德国Merck公司);0.1%十二烷基硫酸钠溶液(北京沃凯生物科技有限公司);超纯水(屈臣氏蒸馏水); 5F-MDMB-PICA、5F-EMB-PICA、ADB-BINACA、AB-PINACA对照品(上海原思标物科技有限公司产品),纯度≥98%。这4种酰胺类合成大麻素的基本信息见表1。

**表1:** 4种酰胺类合成大麻素的具体信息

SC	Chemical formula	Exact mass	Parent ion (m/z)	Daughter ions (m/z)
5F-MDMB-PICA	C_21_H_29_FN_2_O_3_	376.224	377.2	144.1^*^, 232.1
5F-EMB-PICA	C_21_H_29_FN_2_O_3_	376.224	377.2	144.1, 232.1^*^
ADB-BINACA	C_18_H_26_N_4_O_2_	330.213	331.2	201.1^*^, 286.2
AB-PINACA	C_18_H_26_N_4_O_2_	330.213	331.2	215.1^*^, 286.2

* Quantitative ion.

单一标准溶液配制:准确称量5F-MDMB-PICA、5F-EMB-PICA、ADB-BINACA与AB-PINACA这4种合成大麻素的标准物质各5 mg,分别放置于50 mL的容量瓶中,加入甲醇充分溶解并定容,振荡摇匀得到1 mg/mL的标准储备液。用甲醇逐级稀释配制得到1000、500、200、100、50、20、10、5 ng/mL的单一标准溶液备用。

混合标准溶液配制:准确移取质量浓度为1 μg/mL的5F-MDMB-PICA、5F-EMB-PICA、ADB-BINACA与AB-PINACA这4种合成大麻素的单一标准溶液各1 mL,置于10 mL的容量瓶中,加入甲醇充分溶解并定容,用甲醇逐级稀释配制成100、50、25、20、10、5、1 ng/mL的混合标准溶液,振荡摇匀,装入进样瓶备用待测。

### 1.3 检材前处理

毛发检材 将毛发检材用5 mL的0.1%十二烷基硫酸钠溶液、超纯水和丙酮各清洗2次,每次1 min,再用超纯水冲洗3次,去除毛发表面残留的丙酮,放置在阴凉通风处自然晾干。准确称取20 mg清洗干净的干燥毛发检材放置于研磨管中,在研磨管中加入1 mL甲酸-甲醇(1∶2000, v/v)溶液,将研磨管放置于球磨仪中充分混合研磨。将研磨完成的溶液转移至新的研磨管中,放置于超声波清洗器中超声处理30 min。取超声后溶液放置于离心管中,用低温高速冷冻离心机以8000 r/min转速离心10 min,取上清液,过0.22 μm有机微孔过滤膜,装瓶待进一步进样分析。

烟丝检材 称取100 mg烟丝检材放入试管中,加入甲醇溶液10 mL溶解,放置于超声波清洗器中超声处理30 min,取超声后溶液放置于离心管中,用低温高速冷冻离心机以8000 r/min离心10 min,取上清液稀释100倍后进样分析。

### 1.4 实验条件

色谱条件 Hypersil GOLD C_18_色谱柱(100 mm×2.1 mm, 1.9 μm);柱温:30 ℃;流动相A:含0.1%甲酸的10 mmol/L甲酸铵溶液;流动相B: 0.1%甲酸甲醇溶液;流速:0.3 mL/min;进样体积:5 μL;梯度洗脱程序:0~35 min, 50%B; 35~38 min, 95%B; 38~40 min, 50%B。

质谱条件 采用正离子扫描方式;数据采集模式采用一级全扫描(Full MS scan)和数据依赖二级扫描(dd-MS^2^);鞘气压力为38 arb;辅助气压力为10 arb;电喷雾电压为3.8 kV;离子源温度和离子传输管温度均为350 ℃;碰撞气为氩气(Ar)。一级全扫描参数设置:分辨率6×10^4^;最大注射时间100 ms;自动增益控制(automatic gain control, AGC)为3×10^6^;质量扫描范围*m/z* 100~1000。二级扫描参数设置:分辨率3×10^4^;最大注射时间50 ms; AGC为2×10^5^;隔离窗口(isolation window)*m/z* 2.0;归一化碰撞能量(normalized collision energy, NCE)为10、40、70 eV。

## 2 结果与讨论

### 2.1 质谱条件优化

研究过程中共比较了Q Exactive高分辨质谱技术中的平行反应监测(parallel reaction monitoring, PRM)和一级质谱全扫描/数据依赖二级质谱扫描(Full MS/dd-MS^2^)这2种质谱扫描模式。在实验过程中,以5F-MDMB-PICA和5F-EMB-PICA这对吲哚酰胺类合成大麻素同分异构体为例,尝试采用PRM模式对这两种同分异构体进行鉴别区分,但效果无明显改善。由于在公安实践工作中进行物质初筛时,待测分析物通常未知^[1]^,因而PRM模式存在一定缺陷。Full MS/dd-MS^2^则是通过母离子和碎片离子的精确质量数进行定性分析,参考母离子的峰面积进行定量分析,并不要求预知待测目标化合物的相关信息,符合公安实战对于相关酰胺类合成大麻素的快速检验要求。实验最终选择Full MS/dd-MS^2^扫描模式来进行分析物的筛查区分。5F-MDMB-PICA、5F-EMB-PICA、ADB-BINACA与AB-PINACA这4种酰胺类合成大麻素的二级质谱图如图2所示。

**图2:**
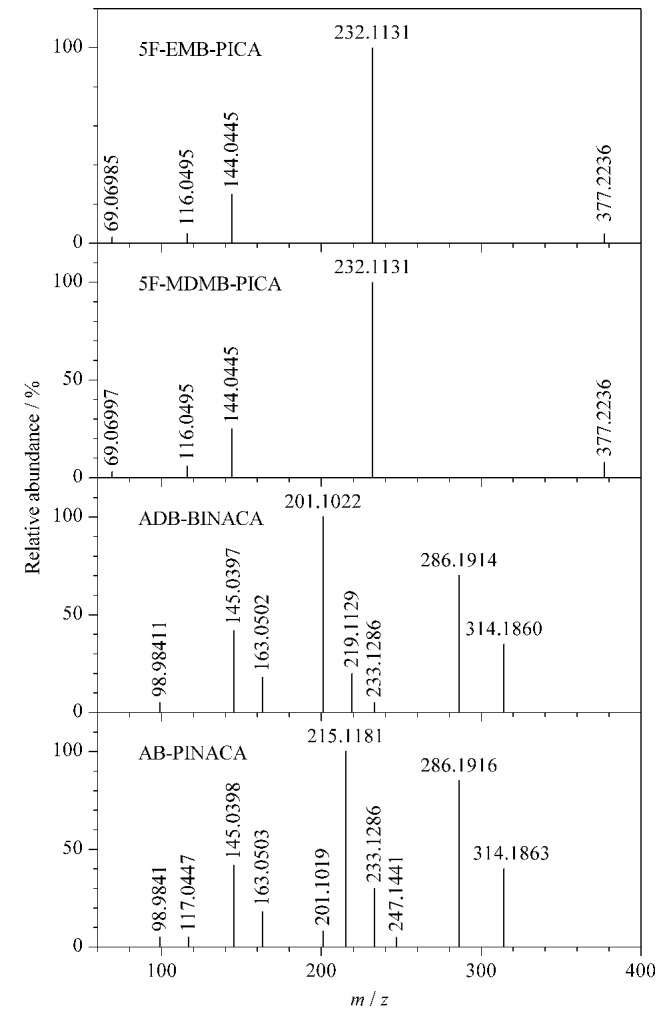
4种合成大麻素在Full MS/dd-MS^2^全扫描模式下的二级质谱图

结合图2和表1中4种酰胺类合成大麻素同分异构体的定性离子对信息,可以发现,5F-MDMB-PICA和5F-EMB-PICA这对同分异构体的两对定性离子对完全相同,均为377.2/232.1和377.2/144.1;而ADB-BINACA和AB-PINACA这对同分异构体的两对定性离子对分别为331.2/286.2与331.2/201.1、331.2/286.2与331.2/215.1,有一对相同的定性离子对。这两对分析物均需要通过优化色谱条件来实现有效区分。

### 2.2 色谱条件优化

#### 2.2.1 流动相条件优化

实验考察了2种有机相,分别是含0.1%甲酸的甲醇溶液和含0.1%甲酸的乙腈溶液;考察了3种水相,分别是0.1%甲酸水溶液、含10 mmol/L甲酸铵的0.1%甲酸水溶液和含10 mmol/L甲酸铵的水溶液。实验结果显示,采用甲醇作为有机相进样时,分析物的响应度要优于乙腈体系,整体峰形更好。此外在有机相和水相中均加入甲酸能够使得基线更为平稳^[20,21]^,在水相体系中加入10 mmol/L的甲酸铵能够有效增强离子强度,抑制化合物解离,显著改善色谱峰形^[22]^。综合上述指标,研究采用含0.1%甲酸的10 mmol/L甲酸铵溶液作为水相,0.1%甲酸的甲醇溶液作为有机相。

#### 2.2.2 梯度洗脱程序优化

参考文献[1],实验初步采用常规梯度洗脱程序对50 ng/mL的5F-MDMB-PICA单标、50 ng/mL的5F-EMB-PICA单标以及50 ng/mL的5F-MDMB-PICA与5F-EMB-PICA混合标准溶液进样分析,得到如图3所示的色谱图结果,可以看出,5F-MDMB-PICA和5F-EMB-PICA这对吲哚酰胺类合成大麻素同分异构体并不能得到有效区分,两者色谱峰发生明显重合。

**图3:**
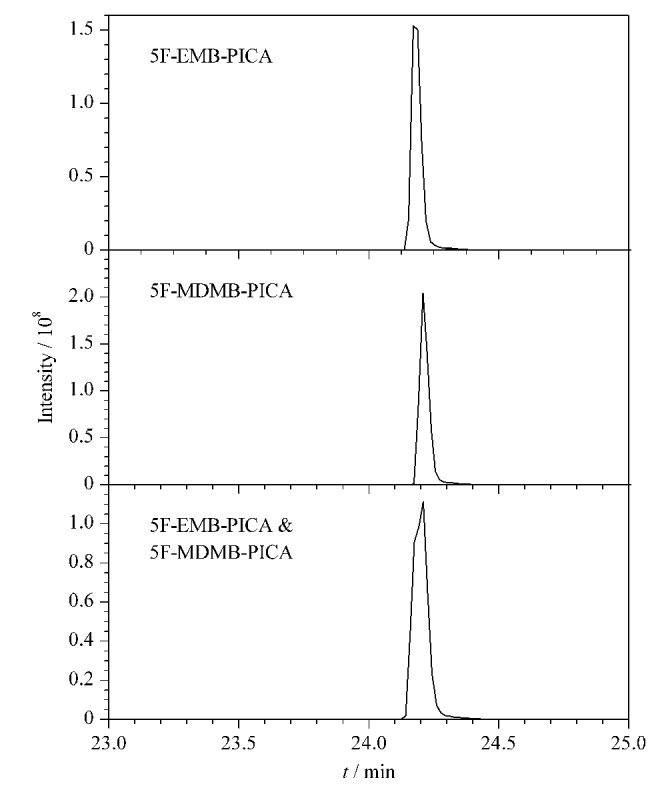
两种合成大麻素单标及混合标准溶液的提取离子色谱图

研究过程中发现,有机相浓度越高,样本物质在流动相的冲洗下出峰越快,相应的同分异构体物质间的出峰间隔越短,而当有机相与水相比例为1∶1时,能有效应对同分异构体间极性差异较小的问题,兼顾分离效果与保留时间效率。在等梯度浓度的流动相作用下,更有利于实现色谱峰完全分离的目的,优化后的洗脱程序参考1.3节。

采用优化的洗脱程序对50 ng/mL的5F-EMB-PICA、5F-MDMB-PICA、ADB-BINACA与AB-PINACA这4种酰胺类合成大麻素的混合标准溶液进样分析,得到的提取离子色谱图见图4。

**图4:**
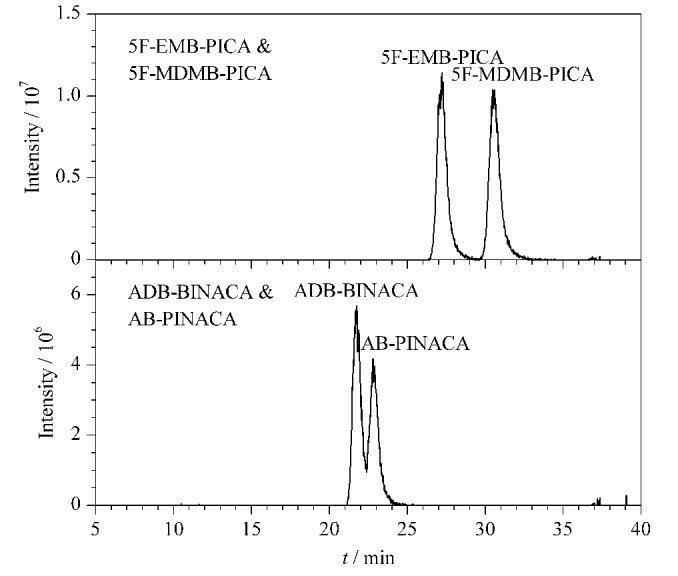
4种合成大麻素的提取离子色谱图

由图4可见,4种酰胺类合成大麻素同分异构体在该色谱条件下均得到了有效区分。在初步采用的洗脱程序条件下,5F-EMB-PICA的保留时间为24.16 min, 5F-MDMB-PICA的保留时间为24.24 min,两者分离度为0.54,远未能达到有效分离的目的。优化后5F-EMB-PICA与5F-MDMB-PICA两者的保留时间分别为27.22 min和30.42 min,分离度达到了2.06,实现了两种分析物的完全分离。ADB-BINACA的保留时间为21.63 min, AB-PINACA的保留时间为23.26 min,分离度为1.22,分离程度达到了98.4%,基本达到了有效分离的目的。结果表明,对4种酰胺类SCs同分异构体采取等梯度方法进样,最终得到高达2.06和1.22的分离度,实现了两对分析物分离的目的,区分效果理想。

### 2.3 线性范围、检出限和定量限

将质量浓度为5、10、20、25、50、75、100 ng/mL的4种SCs混合标准溶液进样分析,每个浓度点均进样3次,以峰面积平均值*y*对质量浓度*x*进行线性回归,结果见表2。以色谱峰的信噪比(*S/N*)≥3,且符合定性要求(即保留时间、峰形及碎片离子丰度比均与相应目标物标准品数据保持一致)时样本的最低浓度作为检出限;以*S/N* ≥10,且满足准确度和精密度要求时的样本最低浓度为定量限^[1]^,结果见表2。结果表明,4种酰胺类SCs同分异构体的线性关系较好,相关系数*R*^2^均大于0.99,能够满足公安实践工作中的检验要求。

**表2:** 4种SCs的线性范围、线性方程、相关系数、检出限和定量限

SC	Linear range/(ng/mL)	Linear regression equation	R^2^	LOD/(ng/mL)	LOQ/(ng/mL)
5F-EMB-PICA	5-100	y=4040966x-1436052	0.9971	1	5
5F-MDMB-PICA	5-100	y=4015304x-8304340	0.9967	1	5
ADB-BINACA	5-100	y=1872702x-915639	0.9965	5	10
AB-PINACA	5-100	y=1167813x-553067	0.9956	5	10

*y*: peak area; *x*: mass concentration, ng/mL.

### 2.4 基质效应、回收率和精密度

当样本为毛发等复杂基质时,基质可能会对分析物的定量分析产生影响,并进一步影响方法的精密度和准确度^[23]^,因此需要对所建方法的基质效应进行考察。分别进样分析低、中、高3个浓度的4种SCs标准溶液、空白毛发样本提取后添加对应浓度的标准溶液、空白毛发样本提取前添加对应浓度的标准溶液。实验通过计算空白毛发样本提取后添加对应浓度的标准溶液得到的峰面积均值与对应标准溶液的峰面积均值的比值来考察基质效应大小。通过计算提取前的空白毛发添加样本与提取后的空白毛发添加样本的对应峰面积均值的比值考察这4种酰胺类SCs同分异构体的回收率。

将平行配制的4种SCs标准溶液分别添加至空白毛发中,制成低、中、高3个水平(0.05、0.5和5 μg/g)的毛发阳性样本,在1天之内的早、中、晚3个时间段分别对同一条件、同一批次下的毛发阳性样本前处理后进样分析^[24]^;通过色谱峰面积的相对标准偏差(RSD)考察精密度指标,每个含量水平平行实验5次,重复上述操作连续进样5天;结果见表3。

**表3:** 毛发样本中4种SCs的基质效应、回收率、日内和日间精密度

SC	Background/ (μg/g)	Matrix effect/%	Recovery (n=5)/%	Intra-day RSD (n=5)/%	Inter-day RSD (n=5)/%
5F-EMB-PICA	0.05	109.52	97.75	5.83	5.41
	0.5	110.26	96.23	4.21	6.13
	5	91.26	100.55	7.12	6.77
5F-MDMB-PICA	0.05	105.76	101.22	3.04	8.12
	0.5	111.76	97.88	6.67	4.32
	5	102.45	99.12	3.46	5.18
ADB-BINACA	0.05	94.76	96.87	6.57	5.54
	0.5	107.24	105.11	7.34	6.98
	5	96.46	102.01	7.83	6.12
AB-PINACA	0.05	88.67	100.29	3.11	2.19
	0.5	105.76	98.79	3.15	7.63
	5	109.23	103.22	2.67	2.51

由表3可以看出,5F-MDMB-PICA、5F-EMB-PICA、ADB-BINACA与AB-PINACA这4种酰胺类SCs在毛发中的基质效应在88.67%~111.76%范围内,表明4种酰胺类SCs在低、中、高浓度水平下进行上述前处理方法的基质效应满足公安实践检测要求,可实现对毛发检材中4种分析物的有效提取与定量分析。5F-EMB-PICA、5F-MDMB-PICA、ADB-BINACA和AB-PINACA这4种SCs的回收率为96.23%~105.11%,日内、日间精密度(RSD)均小于10%,即所建立的该检验技术方法准确度和精密度均良好,符合公安实践中对样本的检验分析要求。

### 2.5 案例应用分析

采用上述建立的UHPLC-HRMS分别对疑似含有合成大麻素的毛发和烟丝检材进行检验鉴定。

毛发和烟丝两份检材通过1.3节中所述方法进行前处理后,均采用UHPLC-HRMS方法检测,得到的结果分别与标准品的相关参数进行对照。检材的色谱图结果见图5。

**图5:**
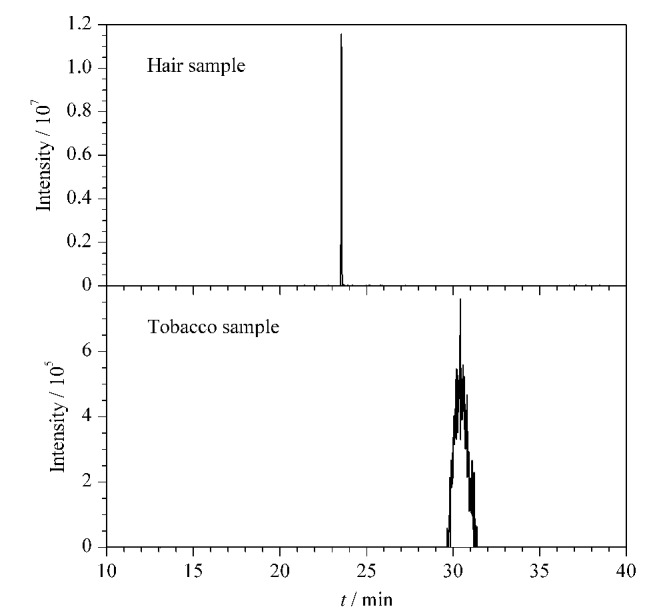
毛发和烟丝样品的提取离子色谱图

如图5所示,两个色谱峰的保留时间分别为23.43 min和30.61 min,与上述AB-PINACA、5F-MDMB-PICA标准品所对应的保留时间一致,结合表1中对应特征离子碎片信息,AB-PINACA的定性离子对为331.2/286.2和331.2/215.1, 5F-MDMB-PICA的定性离子对为377.2/232.1和377.2/144.1,可以确定毛发检材中含有合成大麻素AB-PINACA,烟丝检材中含有合成大麻素5F-MDMB-PICA。

采用外标工作曲线法对毛发检材中的AB-PINACA含量和烟丝检材中的5F-MDMB-PICA含量进行定量分析,得到毛发中AB-PINACA的含量为0.73 μg/g,烟丝中5F-MDMB-PICA的含量为11.3 mg/g。

## 3 结论

本文针对5F-MDMB-PICA和5F-EMB-PICA、ADB-BINACA和AB-PINACA这4种酰胺类SCs同分异构体的区分检验进行了研究,通过优化色谱质谱条件,建立了UHPLC-HRMS定性定量检验方法,成功实现了2对同分异构体的有效区分鉴别,并进行了方法学验证。将本研究建立的UHPLC-HRMS检验SCs同分异构体的技术方法应用于公安实际案例之中,在缴获的烟丝检材中成功检测出5F-MDMB-PICA成分,在毛发检材中检出了AB-PINACA成分,并进行了相应定量分析。该研究成果能够满足公安实战工作中对酰胺类SCs的检验要求,并可为酰胺类SCs中多种同分异构体的检验鉴定提供方法参考与思路借鉴。
